# Give me a sign: decoding four complex hand gestures based on high-density ECoG

**DOI:** 10.1007/s00429-014-0902-x

**Published:** 2014-10-02

**Authors:** M. G. Bleichner, Z. V. Freudenburg, J. M. Jansma, E. J. Aarnoutse, M. J. Vansteensel, N. F. Ramsey

**Affiliations:** 1 UMC Utrecht Brain Center Rudolf Magnus, Utrecht, The Netherlands; 2 Universitair Medisch Centrum Utrecht, Heidelberglaan 100, 3584 CX Huispost: G03.124, Utrecht, The Netherlands

**Keywords:** Electrocorticography, High density, Sign language, Gestures, Decoding

## Abstract

The increasing understanding of human brain functions makes it possible to directly interact with the brain for therapeutic purposes. Implantable brain computer interfaces promise to replace or restore motor functions in patients with partial or complete paralysis. We postulate that neuronal states associated with gestures, as they are used in the finger spelling alphabet of sign languages, provide an excellent signal for implantable brain computer interfaces to restore communication. To test this, we evaluated decodability of four gestures using high-density electrocorticography in two participants. The electrode grids were located subdurally on the hand knob area of the sensorimotor cortex covering a surface of 2.5–5.2 cm^2^. Using a pattern-matching classification approach four types of hand gestures were classified based on their pattern of neuronal activity. In the two participants the gestures were classified with 97 and 74 % accuracy. The high frequencies (>65 Hz) allowed for the best classification results. This proof-of-principle study indicates that the four gestures are associated with a reliable and discriminable spatial representation on a confined area of the sensorimotor cortex. This robust representation on a small area makes hand gestures an interesting control feature for an implantable BCI to restore communication for severely paralyzed people.

## Introduction

With the increasing understanding of human brain function, there is an increasing interest in using that knowledge to interact with the brain to treat brain-related disorders. Electrical stimulation of the brain is used for treatment of movement disorders (Kalia et al. 2013), pain (Boccard et al. 2013) and epilepsy (Fridley et al. 2012), as well as to restore functions such as hearing (Lim et al. 2009) and vision (Normann et al. 2009). Functions can also be restored by recording signals from the central nervous system. The last few decades have seen the emergence of a translational neuroscience field pursuing the goal of restoring or replacing motor function in people with paralysis or lost limbs, using the neuronal activity recorded over the sensorimotor cortex. This approach is referred to as ‘Brain-Computer Interface’ (BCI).

The sensorimotor cortex has been of primary interest for controlling BCI (Pfurtscheller et al. 1993). The underlying idea is to use the neuronal activity of the sensorimotor cortex, which was formerly used for muscle control, for operating an external device. The non-functional peripheral motor system is essentially bypassed. The topographic representation of the sensorimotor cortex (Penfield and Boldrey 1937) conceptually allows for differentiation between movements of different body parts based on neuronal activity.

For decades, scalp electroencephalography (EEG) has been the most widely used technique for BCIs (Wolpaw et al. 2002). More recently, there has been an emergence of intracranial approaches in humans (Zhang et al. 2013). A high degree of robot arm control was achieved in tetraplegic patients using intracranial electrodes (Hochberg et al. 2012; Collinger et al. 2012; Wang et al. 2013). Several studies also have shown that it is possible to decode individual finger (Miller et al. 2009; Kubánek et al. 2009), arm (Ganguly et al. 2009) and complex grasping movements (Pistohl et al. 2012; Chestek et al. 2013) from the sensorimotor cortex in non-paralyzed people using ECoG.

Typically, decoding of movements is pursued for control of robotic arms to manipulate objects (Chestek et al. 2013). However, for severely paralyzed patients who have even lost the ability to speak, communication is the most urgent function that has to be restored. One possibility to achieve this is to use arm movements to control keyboard-like interfaces for communication. Alternatively, arm and hand movements can be also used directly for communication analogs to the way it is done in sign languages, where different hand, arm and body movements have specific meanings. In the finger spelling alphabet, isolated hand movements can be used to represent individual letters of the alphabet. Sign languages, therefore, provide a complete set of hand movements that can be used for communication. Decoding these communicative hand gestures from the sensorimotor cortex could thus provide a ‘cortical alphabet’, where neuronal patterns associated with those movements are translated into letters on a screen or for control of a speech synthesizer (Guenther et al. 2009).

We here test the hypothesis that the topographical organization of the sensorimotor cortex enables reliable identification of sign language hand gestures for communicative BCI. We expand on the results of earlier studies (Pistohl et al. 2012; Chestek et al. 2013) using small high-density electrode grids located pre- and postcentrally on the hand knob area. We have recently shown (Bleichner et al. 2014) that hand gestures can be decoded from a small area of the sensorimotor cortex using high-field fMRI. Given the close correspondence between ECoG and fMRI we have good reasons to believe that the decoding of hand gestures from a small patch of cortex should be equally possible using high-density ECoG (Siero et al. 2013). In this proof-of-principle study we use executed movements in abled-bodied people. Future studies will have to extend this to attempted movements in people who cannot move.

## Methods

### Participants

Two patients implanted with subdural ECoG electrodes for epilepsy diagnostic purposes participated in this study (see Table 1). Data acquisition was approved by the medical ethical board of the University Medical Center Utrecht (UMC Utrecht) in accordance with the declaration of Helsinki (2008). All patients signed informed consent beforehand. Three additional patients were also implanted with high-density grids but the final position of the high-density grids proved to be outside the hand region after closure of the skull. Data from these participants were, therefore, excluded from the study.

**Table 1:** Demographic information, and high-density grid location

	Patient 1	Patient 2
Grid location	Hand knob (pre and post central)	Hand knob (primarily post central)
Number of electrodes	32	60 + 4
Hemisphere	Left	Right
Handedness	Right	Left
Age	19	45
Gender	Female	Female

### Electrodes

The standard electrode grids (2.3 mm exposed surface, inter-electrode distance 1 cm center to center; Ad-Tech, Racine, USA) were placed as usual for clinical purposes. For a small part of the covered area, standard grids were replaced with a high-density grid with 32 or 64 contact points (each with 1.3 mm exposed surface diameter), with an inter-electrode distance of 3 mm center to center (Ad-Tech, Racine, USA). Each electrode measures activity from an estimated 150,000 neurons. The 32-channel high-density grids had a 4 × 8 electrode layout and covered an area of 2.5 cm^2^. The 64-channel grid had an 8 × 8 electrode layout with the four corner electrodes facing the dura, and covered an area of 5.2 cm^2^. We will focus only on the high-density electrode grids.

### Electrode location

**Fig. 1:**
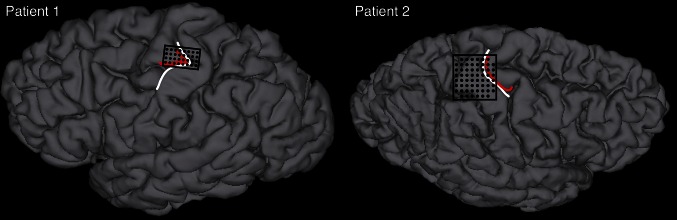
Position of the electrode grid (*black*) shown on the individual anatomy. The *white lines* indicate the central sulcus. The *red lines* indicate the location of the hand knob area, as defined on the axial slices and projected to the surface. For participant 1 the grid was located on the left hemisphere, for participant 2 the grid was located on the right hemisphere

After implantation we checked how far the high-density grids covered the pre- or postcentral part of the hand knob area (Yousry et al. 1997). The electrode locations, acquired with a post-implantation CT were projected on the T1-weighted individual anatomy scan (Hermes et al. 2010). The hand knob area was identified on the axial slices of the T1 scan, by which the individual differences in the shape of the hand knob (Caulo et al. 2007) were taken into account. The location of the hand knob was eventually projected to the surface of the cortex. Two participants completed the study with high-density grids covering most of the hand knob region (Fig. 1).

### Task

**Fig. 2:**
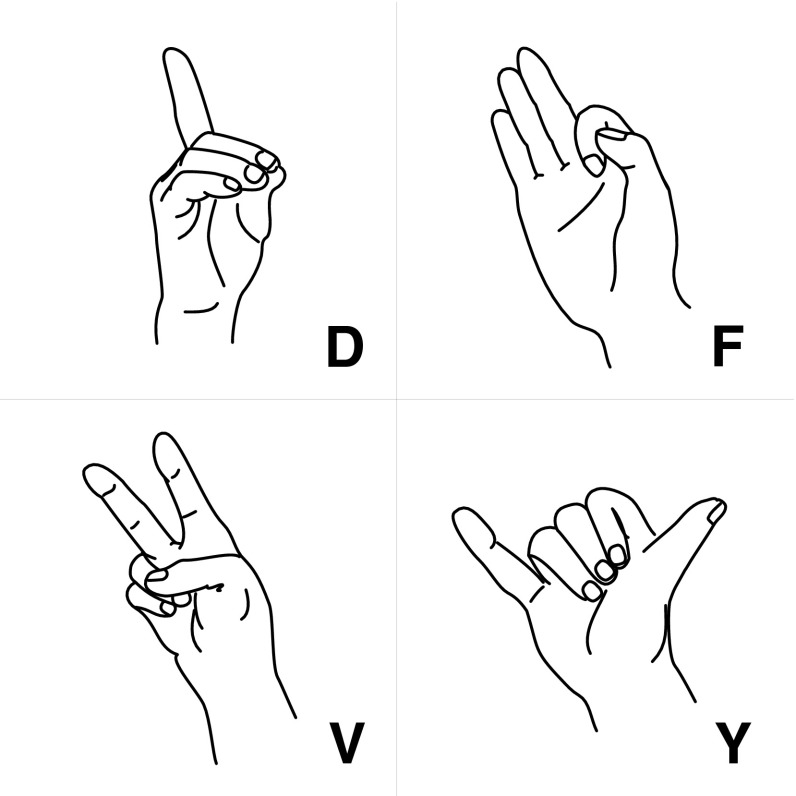
Hand gestures that had to be executed. The gestures differ in the combinations of the fingers that had to be flexed or (kept) extended, and in their similarity with each other. ‘*D*’ and ‘*V*’ are the most alike, as they differ only in the flexion of the middle finger. ‘*D*’ and ‘*F*’ are ‘inverted’ in terms of the fingers that had to be flexed. ‘*Y*’ is different from all other gestures as it is the only gesture that does not require a flexion of the thumb

The participants were asked to execute four hand gestures (depicted in Fig. 2) always starting and ending with a common rest position, i.e. a relaxed open hand. The gestures are taken from the American Sign Language finger spelling alphabet and represent the letters ‘D’, ’F’, ’V’ and ’Y’. The gestures were chosen such that each finger was extended and bent in at least one of the four gestures. The participants were naïve to sign language and, therefore, were briefly familiarized with the four gestures prior to the experiment. Each gesture was presented on the screen and stayed there for 5 s. Participants were asked to copy the depicted gesture immediately after stimulus onset (movement phase I, MP I) and to keep the hand still through the rest of the trial (static phase). Each gesture was followed by a rest condition, in which participants were asked to place their hand back into rest position (movement phase II, MP II). The participants used the hand contralateral to the grid implant. Each gesture was presented 10 times. Participant 2 performed the task two times.

### Task performance

The actual hand gestures were recorded using a data glove (5 DT Inc, Irvine, USA). This data glove provided measurements of the bending movement for each finger. The data glove measurements were inspected visually for correct bending of the fingers, and for absence of additional movements. Erroneous trials were excluded from further analysis, i.e. those in which the gesture was executed incorrectly, or when additional fingers were moved, or when a correction of the gesture was necessary. The movement onset was determined for each trial based on the first deflection from baseline for one of the fingers that lead to the execution of the gesture.

### ECoG preprocessing

Signals were recorded continuously using a 128-channel Micromed (Treviso, Italy) system (22 bits, band pass filter 0.15–134.4 Hz) at a sampling frequency of 512 Hz. Offline, the data were band-pass filtered to exclude the 50 Hz line noise and re-referenced to the common average of all electrodes of the high-density grid.

The data were aligned to movement onset as determined by the data glove and epoched into segments of 10 s, using the interval from −2 to 8 s around movement onset. The interval from −2 to −1 s with respect to movement onset was used as baseline. The mean power of five frequency bands (4–8, 8–14, 15–30, 65–95, 70–125 Hz) similar to the ones used in Kubánek et al. (2009) was computed for each electrode. Additionally the local motor potential (LMP) was computed as it was shown to allow for good movement discrimination (Schalk et al. 2007). For classification the average band power (amplitude for the LMP) of a 3 s segment of the first movement phase (from the interval −1–2 s around movement onset) was used.

Based on previous studies we expected the frequencies above 65 Hz to be most informative to discriminate the individual gestures (Miller et al. 2009; Kubánek et al. 2009; Hermes et al. 2012; Chestek et al. 2013). Due to limitations of the recording system frequencies above 130 Hz could not be considered in the analysis.

### Classification

The single trial data were classified using a pattern (template) correlation approach with a leave-one-out cross-validation scheme (Haxby et al. 2001; Misaki et al. 2010). The classification was performed separately for each frequency band. The used feature set was the averaged power in the given frequency band per electrode; for the LMP the average amplitude was used instead. This resulted in a 1 × 32 or 1 × 60 (depending on grid size) feature vector per trial.

For each gesture the average activation pattern (called prototype from here on) was computed over trials. The single trial that was to be classified was left out of the corresponding average. The single trial feature vector was consequently correlated with the four prototypes using Pearson correlation. The single trial was labeled as the gesture (prototype) it had the highest correlation score with. This was repeated for each trial. The performance metric was the number of correctly classified trials (given as percentage). Confusion matrices were constructed to provide information about the type of errors made.

To further validate the classification results, a regularized latent discriminant analysis (rLDA) was also applied to the data, another frequently used classification method (Misaki et al. 2010; Blankertz et al. 2011). For this we used the MATLAB extension BCILAB (Kothe and Makeig 2013), with the same feature set as was used for the pattern correlation analysis.

### Statistical threshold

The theoretical chance level of 25 % for classification of four classes might not be accurate, due to the small and unbalanced number of trials. We, therefore, computed an empirical significance level. For this the classification accuracy was re-computed using randomized labels in 10,000 permutations. From the resulting distribution the mean and standard deviations were computed. The significance threshold for our classification results was three times this standard deviation above the calculated mean.

### Minimum number of electrodes and most informative electrodes

To get an estimate of the minimum number of electrodes required to achieve optimal classification accuracy and to get an estimate of the relative contribution of the individual electrodes, the following procedure was applied: the classification accuracy was re-computed with decreasing numbers of electrodes; the set size varied between all electrodes to an individual electrode. For each set size the classification was computed using random combinations of electrodes. It was assured that each electrode was present in at least 400 combinations. This lead to 12,800 random electrode combinations for the 32-electrode grid and 24,000 different electrode combinations for the 64 electrode grid. For the set sizes that had less than 12,800 or 24,000 electrode combinations, respectively, all possible electrode combinations were used. The contribution of each individual electrode was computed based on the average classification that was achieved when that electrode was part of the combination.

### Temporal information

For more insight into the temporal information in the data the classification accuracy was re-computed for different intervals using a moving window of 1 s for the interval from −1.5 to 8 s around movement onset.

### Template similarity and classification confidence

The Pearson correlation of the templates with each other was computed to get an estimate of the similarity of the templates. For each split the correlation between templates was computed and subsequently averaged over splits. Furthermore, the average correlation score of the individual trials with the corresponding template (including only correctly classified trials) and non-corresponding templates (including only correct rejections) was computed. The difference of the correlation scores between hits and correct rejections was expressed as percentage difference.

### Task activity

For each electrode and frequency band it was determined whether there was a significant increase in power during the first movement phase, compared to the pre-movement baseline (Pearson correlation, alpha level 0.01). For comparing the differences in power between the first and second movement phase, a within trial paired *t*-test on the maximal power within the two movement segments was computed. For comparing power differences between gestures during the first movement phase a one-way between conditions ANOVA was conducted per electrode and frequency band.

## Results

**Fig. 3:**
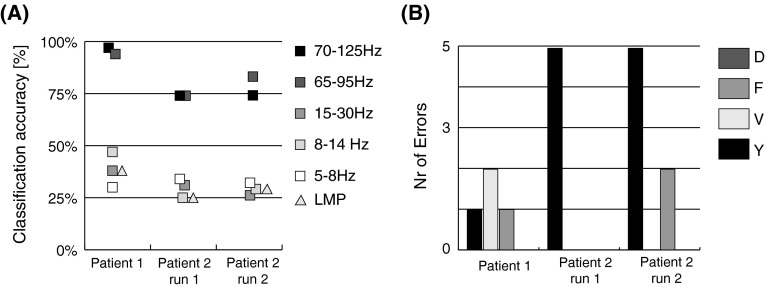
**a** Classification accuracies for five frequency bands and the local motor potential (LMP) for the individual datasets. The empirically determined significance level lay around 50 % for both participants. The high frequencies (>65 Hz) show good classification results. The low frequencies (<30 Hz) and the LMP are consistently at or just above chance level. The classification accuracies are consistent between the first and second run of participant 2. **b** Number of execution errors per gesture and participant. Participant 2 had problems with performing ‘*D*’ in both runs. Incorrectly executed gestures were excluded from analysis

For both participants the classification scores were above the empirically determined significant threshold (around 50 % for all three datasets) for the high frequencies (>65 Hz). The classification scores for the low frequencies (<30 Hz) and the LMP were at or just above chance level (Fig. 3a). This indicates that the grid activation patterns at low frequencies do not offer discrimination between the gestures on this spatial scale. In the following the results are presented for each participant individually for the high frequencies.

### Participant 1

The location of the high-density grid of participant 1 corresponded optimally with the anatomical location of the hand knob, covering pre- and postcentral areas to equal extents (Fig. 1). The gestures were executed with few errors (Fig. 3b).

**Fig. 4:**
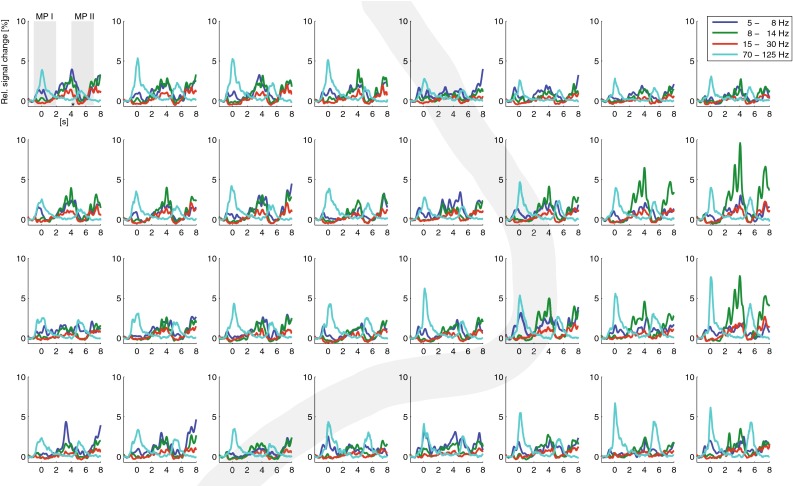
Band specific power (shown for four frequencies bands) over time relative to pre-movement baseline averaged over all gestures for each electrode; shown for participant 1. Electrodes are arranged according to their position on the grid. The *thick grey line* indicates the central sulcus. Movement phase I (MP1, first *shaded grey area*) and movement phase II (MP 2, second *shaded grey area*) are indicated for one electrode

Averaged over all gestures there were clear band-specific responses during hand movement. The high frequencies showed a clear increase in power during the two movement phases. All (but one) electrodes showed a significant movement-related change in power between the MP I and the pre-movement baseline. For most electrodes the signal change during MP I was significantly larger compared to MP II. During the static phase in which the hand stayed in the gesture position the power in the high frequencies went back to baseline level. The lower frequencies were clearly suppressed during both movement periods (Fig. 4).

**Fig. 5:**
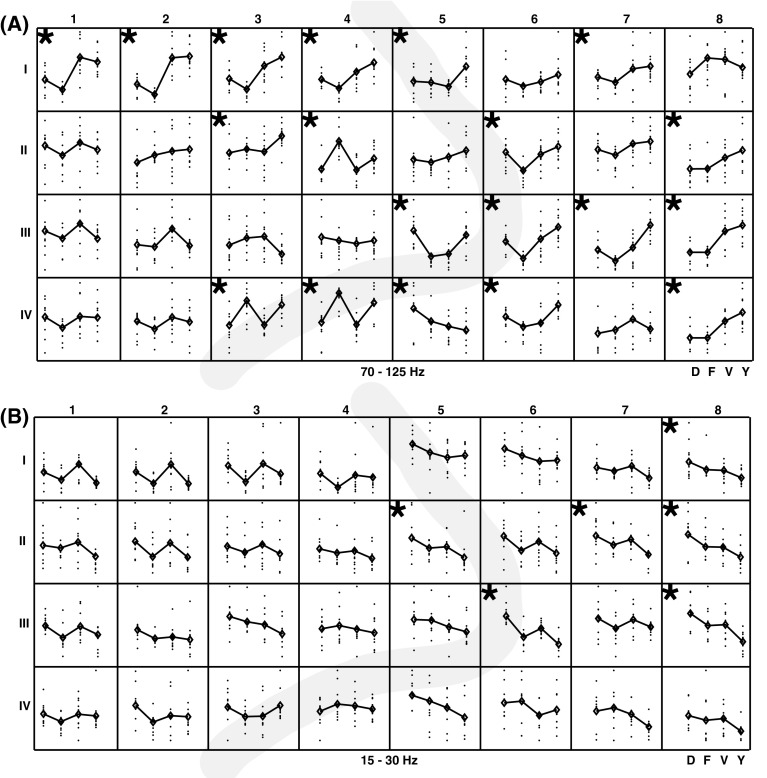
Averaged band power of the 70-125 Hz band (**a**) and the 15–30 Hz band (**b**) for each electrode arranged according to their position on the grid; shown for participant 1. For each gesture the power of the individual trials (*black dots*) and the mean over trials (*black diamond*) are shown. The *black star* in the *upper left* *corner* indicates a significant difference between conditions (*p* < 0.01)

There were clear differences in power between the individual gestures for the high frequencies. Two-thirds of the electrodes (20 out of 32) showed significant (*p* < 0.01) differences between the four gestures (based on a one-way ANOVA). Approximately one half of those electrodes was located on the motor cortex and half on the sensory cortex (Fig. 5a). Some individual electrodes could perfectly discriminate between different gestures for the high frequency band (e.g. I1 and III5). It is readily apparent that based on for instance electrode I1 (as well as electrodes I2 and I3) ‘F’ and ‘Y’ could be discriminated perfectly. Electrode III5 on the other hand allowed to discriminate between ‘D’ and ‘F’. Neighboring electrodes sometimes showed the same preferences, but sometimes exhibited completely different patterns (e.g. Fig. 5a: electrodes I2 and I3 were similar but I3 and II4 were different). This suggests that the electrode distance was not too small and electrode signals (at least 70–125 Hz) were not correlated with each other. Similar patterns were the result of similar behavior of the neural ensembles underneath electrodes. There were no electrodes that are specific to only one gesture, i.e. for differentiating between four gestures the combination of multiple electrodes were necessary. For the lower frequencies (e.g. 15–30 Hz) fewer electrodes showed a significant difference between conditions (6 out of 32).

**Fig. 6:**
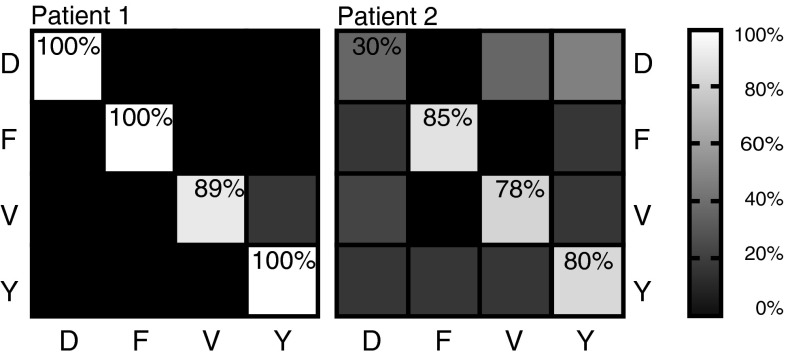
Average confusion matrix showing percent classification rate for each of the gestures for the results of the 70–125 Hz range. For participant 2 the average classification matrix of the first and second session is shown. The classification percentage is shown as *grey* values as indicated by the bar on the right. The *vertical axis* shows the actual label, the horizontal axis shows the predicted label. Perfect classification is a *white* diagonal from *upper left* *corner* to lower *left corner*. For correct classifications the score is also indicated in each field. Classification was almost perfect for participant 1. For participant 2 the classification accuracy of ‘*D*’ was low. Most classification errors made were either ‘*D*’ trials that were classified as ‘*V*’ or ‘*Y*’, or trials of all other conditions that were misclassified as ‘*D*’

**Fig. 7:**
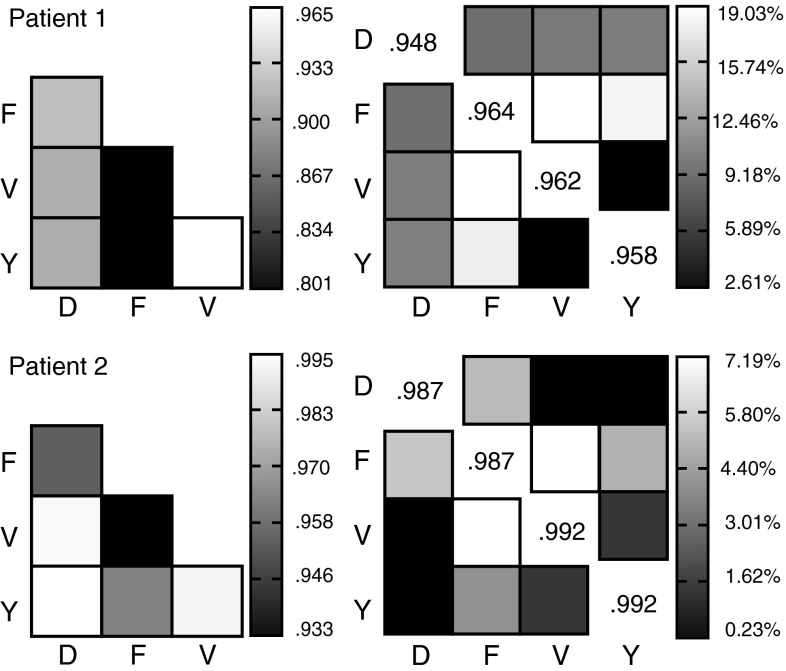
*Left column*: similarity of templates with each other expressed as Pearson correlation. For both participants ‘*Y*’ and ‘*V*’ are most similar to each other, while ‘*F*’ is the most different from all others. For participant 2 there is a high correlation of ‘*D*’ with ‘*Y*’ and ‘*V*’ which explains the misclassifications. *Right column*: similarity of individual trials with templates. The diagonal shows the average correlation coefficient of the individual trial with the corresponding template (i.e. correct classifications). Off- diagonal is the percentage difference of the correlation coefficient with the not corresponding templates

The classification accuracy was 97 % (91 % for rLDA) using the 70–125 Hz band (Fig. 3a). There were no differences in classification accuracies between the gesture types (Fig. 6). In one case a ‘V’ was misclassified as a ‘Y’. On average the templates were strongly correlated with each other (*r* = 0.88). The strongest correlation was between ‘V’ and ‘Y’; the lowest correlations were between ‘F’ and all other gestures (Fig. 7, left column). Figure 7 (right column) shows the percent difference of the correlation scores between the trial and the corresponding template and the non-corresponding templates. The individual trials correlated highly with their corresponding templates (i.e. correct classifications). The ‘F’ trials were classified with the highest confidence. For ‘V’ and ‘Y’ trials the difference in the correlation score was only 2.5 %. Despite the small difference in the correlation scores the classifications were still consistently correct.

Some of the electrodes (located pre-and postcentrally) were more informative for the classification allowing to differentiate between gestures directly (e.g. electrode I5, III3 and III8 on Fig. 5a).

**Fig. 8:**
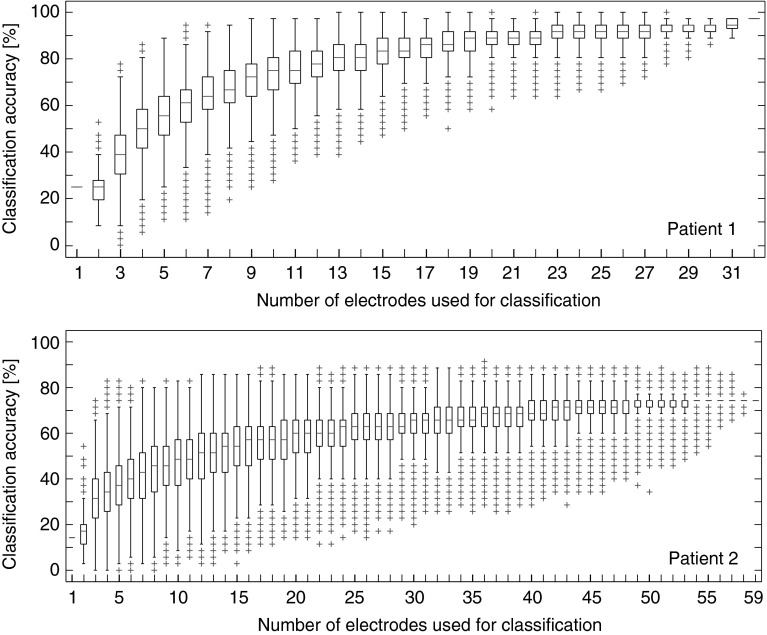
Classification accuracy for variable number of electrodes used for classification shown for participant 1 (*top*) and 2 (*bottom*, first run shown) for the results of the 70–125 Hz range. For each number of electrodes the classification accuracy for a random selection of electrodes was computed. Shown is the median classification score reached for the set size, the edges of the *box* are the 25th and 75th percentiles, the whiskers extend to the most extreme data points not considered outliers, and outliers are plotted individually (cross). Classification accuracies increase on average with the number of included electrodes. As indicated by the whiskers, there is a large variability in classification accuracy depending on the selection of electrodes. There are some combinations of electrodes that allow classification rates as high or even higher than using the total number of electrodes. This indicates that some electrodes are more informative than other, that optimal classification rates can be achieved with a subset of electrodes and even that some electrodes can be detrimental for the classification accuracy

Averaged over the different combinations of electrodes the highest classification scores were reached with all 32 electrodes (Fig. 8). However, there were some combinations of electrodes that allowed comparably high or even higher classification accuracy with less electrodes (e.g. see whiskers for most of the set sizes).

**Fig. 9:**
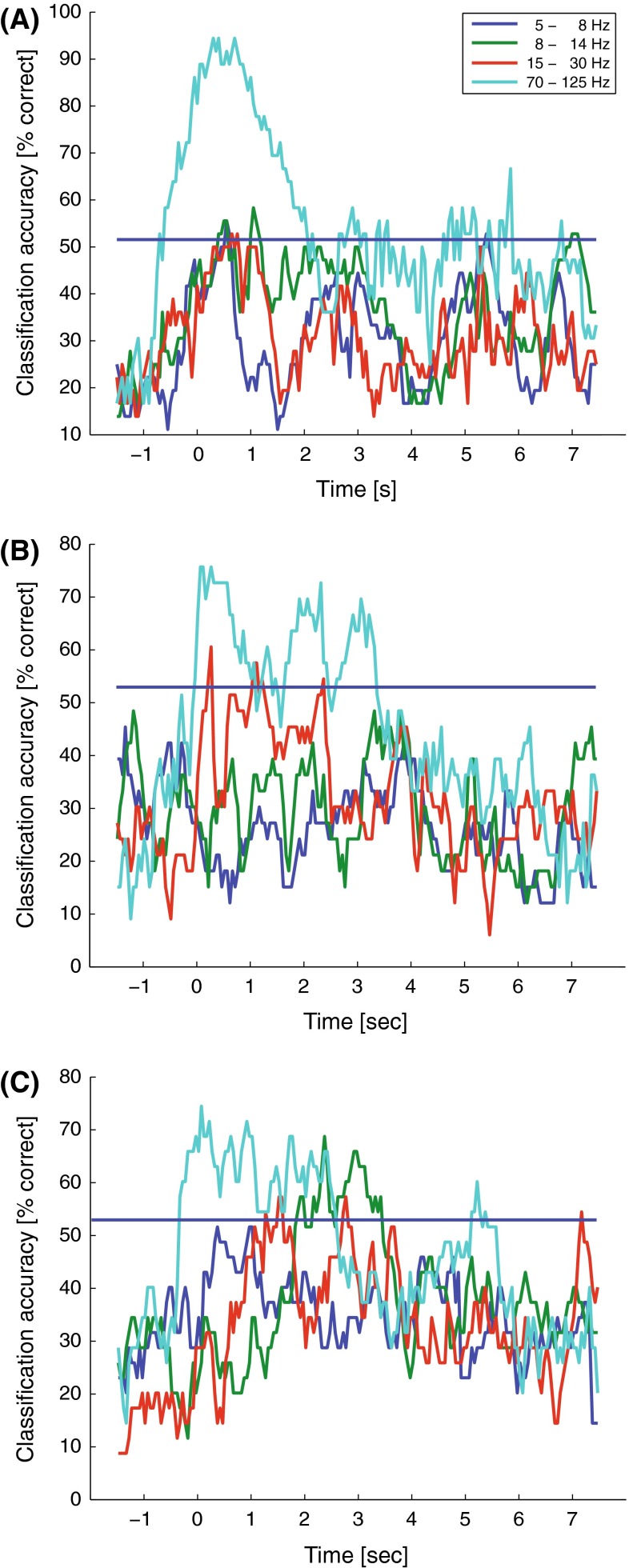
Classification accuracy over time for participant 1 (**a**) and participant 2 (**b**, **c**) shown for four frequency bands. Each data point represents the classification accuracy of a one second segment (centered at that time point). The blue horizontal bar indicates the empirically determined significance level. Time zero is movement onset. The most informative period is the time at and after movement onset. The high frequencies are the most informative throughout the entire period

The most informative period was at and around movement onset from rest to gesture position (see Fig. 9a). Only the high frequencies (70–125 Hz) surpassed the significance threshold reliably. During the static phase (2–4 s) and the second movement phase the classification accuracy is low.

### Participant 2

For participant 2, the actual grid location corresponded reasonably well with the anatomical location of the hand knob, although the grid was primarily located on the sensory cortex (Fig. 1). This participant performed two sessions. In both sessions the participant had great problems executing the ‘D’ gesture and, therefore, half the ‘D’ trials were excluded (Fig. 3b). Furthermore the participant moved in some trials during the static phase instead of keeping the gesture position throughout the trial.

**Fig. 10:**
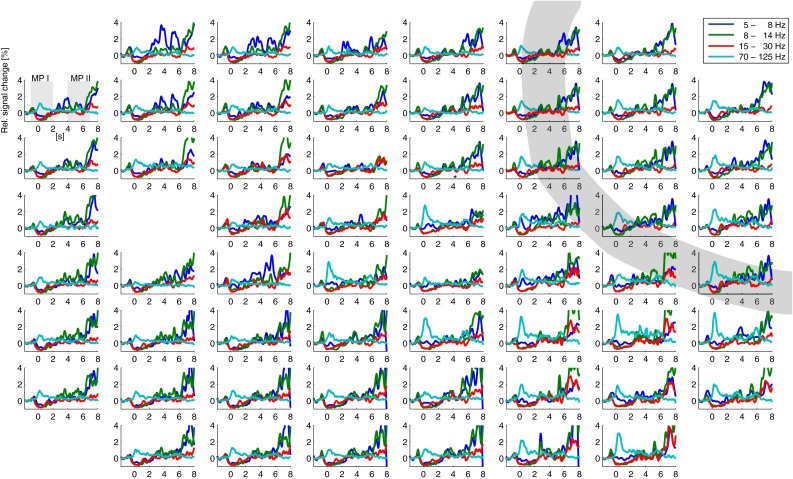
Band specific power (shown for four frequencies bands) over time relative to pre-movement baseline averaged over all gestures for each electrode; shown for participant 2. Electrodes are arranged according to their position on the grid. The *thick grey line* indicates the central sulcus. Movement phase one (MP1, first *shaded grey area*) and movement phase two (MP 2, second *shaded grey area*) are indicated for one electrode

Averaged over all gestures there were clear band-specific responses during hand movement. The high frequencies show a clear increase in power during the two movement phases. All (but one) electrodes showed a significant movement-related change in power between the MP I and the pre-movement baseline. For half of the electrodes the signal change during MPI was significantly larger compared to MP II. The lower frequencies were clearly suppressed during both movement periods (Fig. 10).

**Fig. 11:**
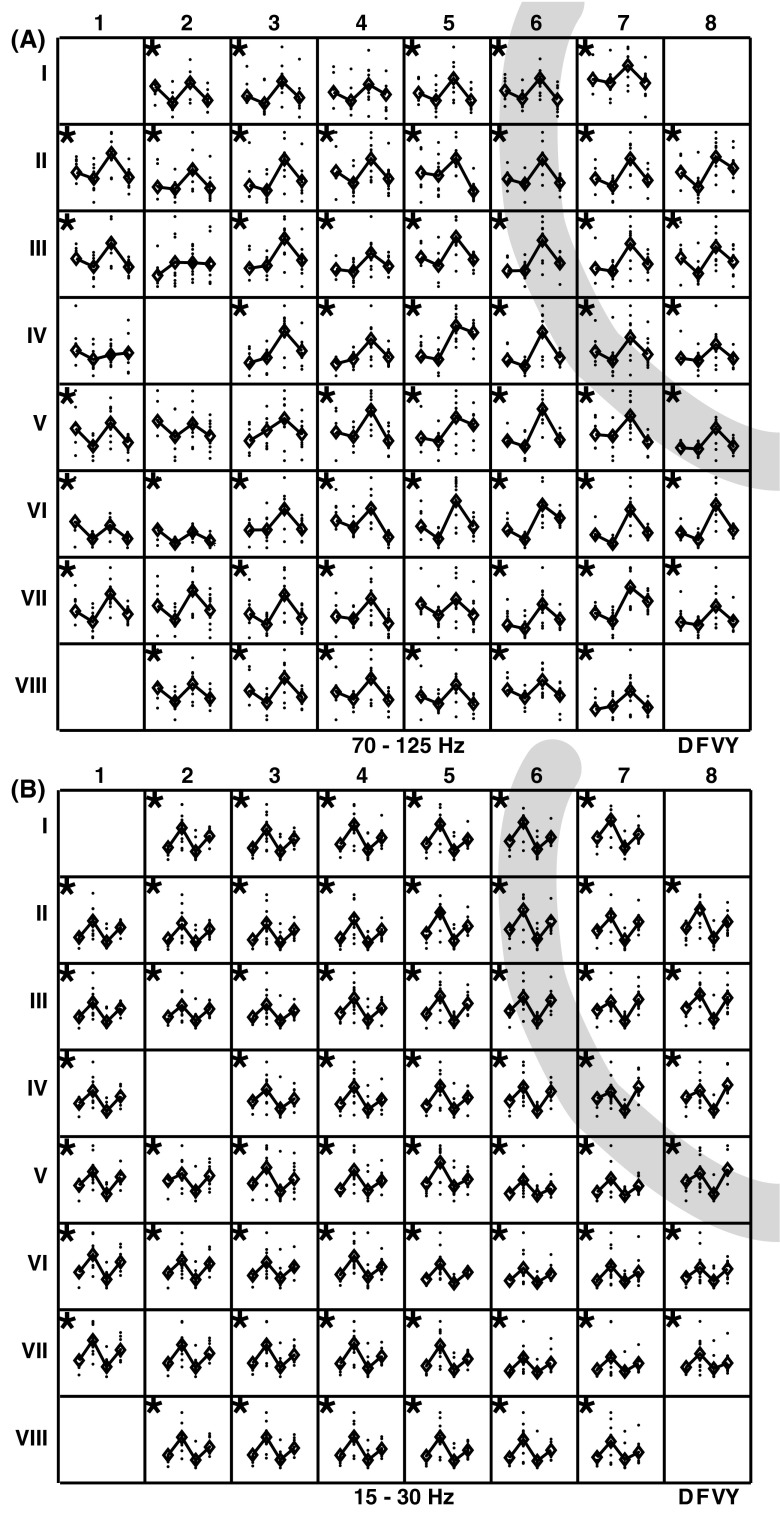
Averaged band power of the 70–125 Hz band (**a**) and the 15–30 Hz band (**b**) for each electrode arranged according to their position on the grid; shown for participant 2. For each gesture the power of the individual trials (*black dots*) and the mean over trials (*black diamond*) are shown. The *black star* in the *upper left* *corner* indicates a significant difference between conditions (*p* < 0.01)

The majority of electrodes showed significant differences between the four gestures (based on a one-way ANOVA) for the high frequencies. Some individual electrodes could perfectly discriminate between different gestures for the high frequency band (e.g. VI 6, Fig. 11a). However, the differences between gestures were less pronounced compared to participant 1. Overall the activation patterns are more alike. There were no electrodes that were specific to only one gesture. For the lower frequencies (e.g. 15–30 Hz) the differences between gestures all point in the same direction (Fig. 11b).

The frequency band 70–125 Hz allowed for 72 and 74 % (68 and 78 % for rLDA) average classification for the two datasets, respectively (Fig. 3a). The problems with executing the ‘D’ gesture were also reflected in the classification scores for the individual gestures. The ‘D’ gesture was classified around chance level while the other three gestures were classified with an accuracy of ~80 % (Fig. 6). Most of the errors made were either misclassification of ‘D’ as ‘V’ or ‘Y’ or misclassifications of the other gestures as ‘D’.

On average the templates were strongly correlated with each other (*r* = 0.97). The strongest correlation was between ‘D’, ‘V’ and ‘Y’; the lowest correlations were between ‘F’ and all other gestures (Fig. 7, left column). Figure 7 (right column) shows the percent difference of the correlation score between the trial and the corresponding template and the non- corresponding templates. The individual trials correlated highly with their corresponding templates (i.e. correct classifications). The ‘F’ trials were classified with the highest confidence. For ‘V’ and ‘Y’ the difference in the correlation score was only 1 % and only 0.5 % for ‘D’ and ‘Y’, and ‘D’ and ‘V’. This explains the misclassifications between those gestures.

On average the highest classification scores were reached with all 59 electrodes (Fig. 8). However, there were some combinations of electrodes that allowed comparably high or even higher classification accuracy with less electrodes (e.g. see whiskers for most of the set sizes).

The most informative period was at and around movement onset (see Fig. 9b, c). Only the high frequencies (70–125 Hz) surpassed the significance threshold reliably for both datasets. During the static phase (2–4 s) and the second movement phase the classification accuracy was low.

## Discussion

We have shown in this study that it is possible to distinguish four hand gestures with high accuracy from the sensorimotor cortex using high-density subdural ECoG grids with electrodes that each measure from neuronal populations on the order of 100,000–150,000 neurons (Shepherd 2003). Participant 1 achieved a 97 % accuracy and participant 2 a 72–74 % accuracy (in two sessions).

For both participants we found that the high frequencies (>65 Hz) gave the highest classification accuracies which is in line with previous research (Pistohl et al. 2012; Chestek et al. 2013). The low frequencies that are commonly used for EEG-based BCI as well as the LMP used to differentiate arm movements using ECoG (Schalk et al. 2007) did not allow to discriminate the four gestures. (Also the combination of low frequencies with high frequencies (data not show) did not lead to higher classification accuracies in comparison to only using the high frequencies.)

With this finding, we extend the existing literature by showing that classification is possible using a confined patch of cortex (the high-density grid covered a small surface of 2.5–5.2 cm^2^, 32 electrodes and 64 electrodes, respectively) over the hand knob area.

In both participants we found that almost all electrodes showed significant activity for at least one gesture. A subset of at most two-thirds of the electrodes could have sufficed to reach similar classification rates as those obtained with all electrodes. This suggests that eventually a smaller number of electrodes can be implanted. The challenge will be to determine where the electrodes should be prior to implantation.

During the two movement phases there were clear differences in power relative to baseline. During the static phase the power in the high frequencies went back to baseline for both the precentral as well as the postcentral electrodes. This indicates that the high frequency response is specific for the movement phase itself and less for keeping the fingers in their position. The power difference between MP I and MP II is likely due to the complexity of the movement. While making the gestures during MPI requires a coordinated action to get all fingers into the right position, moving back to the baseline condition during MP II can be achieved by simply relaxing the hand. Decoding of the gestures was only possible with high accuracies during MP I. It is apparent that there are no gesture-specific electrodes (differentiating one gesture from all others) for both participants, and that neighboring electrodes can show distinct activation patterns.

While participant 1 performed with 97 % accuracy above the 90 % criterion that is requested by potential BCI users (Huggins et al. 2011), participant 2 did not (with maximal 74 %). Participant 2 appeared to have problems performing one of the gestures (‘D’). However, incorrectly executed trials were excluded from the analysis and should, therefore, not have an effect on the overall classification rate. When ‘D’ was excluded completely from the analysis (data not shown) the classification was 85 and 86 %. Compared to participant 1, participant 2 had considerably higher correlation scores between the templates indicating greater similarity between the neural representations. The gestures that correlated strongest (‘D’ with ‘Y’ and ‘D’ with ‘V’) were misclassified most often. A possible explanation for the high similarity of the neuronal representation of the gestures in participant 2 is the location of the grid on the postcentral sulcus. While it has been shown that postcentral gyrus close to the central sulcus does play a role in motor function (Uematsu et al. 1992), most of the electrodes in participant 2 were located over the sensory areas. Based on the work of Sanchez-Panchuelo et al. (2012) one could expect clear separability of the gestures based on their differences in sensory feedback (i.e. for each gesture a different set of finger segments is touched). However, there are a couple of factors that interfere with normal sensory input in our experiment. First, we do not know how much sensory feedback the participants received, as we do not know with how much force the gestures were executed. Second, the fabric of the dataglove causes unspecific sensory feedback over the entire hand during each movement. Finally, the dataglove was not tight fitting, hampering sensory feedback especially at the finger tips due to excess material. Taken together these factors might explain the lower discriminability of the gestures to some degree.

Interestingly, we found some consistency in the representation of the gestures between the two participants. For both participants the ‘Y’ and ‘V’ gestures were the most alike in terms of their neuronal activity, despite the fact that they vary considerably in the combination of fingers that had to be flexed (Fig. 2). Furthermore, the ‘F’ gesture was the most different from the other gestures in both participants. Despite the overall high correlation scores between the templates the trials were sufficiently and consistently different from each other to be discriminated.

Due to the limited time period available with the participants it was not possible to acquire more trials or to test a larger variety of hand gestures. Nevertheless, there do not seem to be any methodological limitations to extend the number of gestures. Chestek et al. (2013) showed in their study (albeit with mostly standard grids and covering multiple brain regions) that nine different grasping movements could be discriminated with high accuracy. Therefore, it can be assumed that more gestures can be discriminated.

The high classification scores that were reached using a simple pattern correlation classification (confirmed by rLDA) show that the underlying neuronal patterns are highly stable and reproducible, at least for the duration of the experiment.

The results presented here extend our previous findings where we have shown that four hand gestures could be differentiated using high-field fMRI (Bleichner et al. 2014). The size and location of cortex used for classification was comparable in both studies. By showing that classification is also possible using subdural electrode grids we have taken the next step towards using hand gestures as control signals for an implantable BCI system for paralyzed patients to re-establish communication.

We argue here that the topographical organization of the hand in the sensorimotor cortex provides a control signal with many important advantageous characteristics for implantable communicative BCIs. Sign language provides in principle a complete set of sufficiently distinct gestures that can serve to communicate. Ideally, if gestures corresponding to the entire alphabet could be identified using the current method, it would allow a speech-like control signal.

Importantly, we have shown here that a small patch of cortex, covered with high-density grids, is sufficient to decode hand movements to a very promising degree of accuracy and thereby extend the findings by Chestek et al. (2013). Using high-density instead of standard ECoG grids facilitates a minimally invasive BCI (Zhang et al. 2013), with a number of benefits for the patient. Implanting high-density grids, over a previously identified target area allows for smaller, minimally invasive surgeries, thereby leading to shorter hospitalization and reduction of risks and complications, such as epileptic seizures, leakage of cerebrospinal fluid, infection, scarification and cosmetic consideration (Reisch et al. 2013).

In comparison to needle electrodes or microwires, the average signal of a population of neurons, as measured with ECoG, may be expected to be more stable over time and thereby require less re-calibration. This needs to be verified in further studies. The electrode spacing of 3 mm (center to center), allows for recording distinctive signals from neighboring electrodes, which makes it possible to exploit the fine-grained organization of the sensorimotor cortex (Sanes et al. 1995; Schieber 2001).

One of the major possible drawbacks of a BCI can be that the control signal interferes with other tasks that the BCI user wants to perform (Ramsey et al. 2004). There are several characteristics of our approach that limit interference with other cognitive tasks. First, use of gestures as a means of communication can become automatic, as demonstrated by for instance deaf people who use it on a daily base. Our approach is self-paced, and thus does not require the user to pay attention to externally timed stimuli. This makes it also interesting for visually impaired patients who are incapable of controlling their eye gaze (Brunner et al. 2010).

In a previous study we have demonstrated a close correspondence between fMRI measurements and ECoG data (Hermes et al. 2012; Siero et al. 2013). Those results indicate that it is possible to optimize the ECoG grid position with fMRI prior to implantation. The correspondence between fMRI and ECoG also indicates that participants can be trained to control a BCI using an fMRI feedback task prior to electrode implantation. This would ensure that the patient is capable of performing the task and to learn to control a BCI before he undergoes the risk of a surgery.

Before the current approach can be used to help paralyzed people, additional steps have to be taken. First, the observed differences between the first and second dataset make it necessary to determine precisely the importance of the localization of the electrodes in functional terms. Second, it needs to be shown that the results presented here using executed movements also hold for paralyzed patients. Obviously, paralyzed patients are incapable of executed movements. For controlling a BCI using gestures they have to either imagine (i.e. think to perform the movement) or attempt (i.e. try to perform the movement) the corresponding movements. Whether imagined or attempted gestures can be decoded in paralyzed patients remains to be shown.

We have, however, good reasons to believe that it is also possible to decode attempted gestures in paralyzed patients. There is hemodynamic and electrophysiological evidence that the general topographic representation of the primary motor cortex is largely preserved in tetraplegics after extensive periods of paralysis (Shoham et al. 2001; Corbetta et al. 2002; Sabbah et al. 2002; Cramer et al. 2005; Hotz-Boendermaker et al. 2008; Mattia et al. 2009, but see also Yanagisawa et al. 2012).

Furthermore, it has been shown that attempted movements provide a successful control strategy for BCI. Several studies (Hochberg et al. 2012; Collinger et al. 2012; Wang et al. 2013) have shown that the sensorimotor cortex of paralyzed patients provides sufficient information to control a robotic arm in several dimensions using attempted movements. Blokland et al. (2012) have shown that tetraplegic patients have a better BCI control using attempted instead of imagined movements.

Given that executed and attempted movements show a higher resemblance in terms of their pattern of activation (Sabbah et al. 2002) than imagined and attempted movements, we expect our results to generalize to some degree to paralyzed patients.

Third, several practical issues need to be solved before our approach can be taken to paralyzed patients. For efficient communication it is necessary that gestures following in fast succession can still be discriminated. Also, the event of false alarms, where a gesture is detected despite the fact that the user did not intend to send that signal, needs to be minimized. Finally, there are also several important limitations that currently prevent a completely implantable system based on intracranial electrodes. At this moment, there are no implantable systems on the market that are approved for human use that allow the simultaneous pre-amplification and wireless transmission of large numbers of channels. Consequently, it is necessary to keep the number of channels limited, finding a tradeoff between the discriminative power and the feasibility in terms of signal processing and transmission.

## Conclusion

Brain activity patterns generated by four different hand gestures can be distinguished from a small region of the sensorimotor cortex. The results of this proof-of-principle study indicate feasibility of decoding multiple control states from a small patch of cortex for intracranial BCI. The optimal location of the electrode grid may be determined a priori using high-field fMRI and anatomical landmarks. Although only four gestures were tested, the high classification rate suggests that good results may be obtained for larger numbers of gestures when decoding from this region, bringing the concept of directly decoding internal spelling and of a ‘cortical alphabet’ for BCI closer.
